# VCAM1 and ICAM1 expression in oral lichen planus

**Published:** 2013

**Authors:** Maryam Seyedmajidi, Shahryar Shafaee, Ali Bijani, Soodabeh Bagheri

**Affiliations:** 1 *Dental Materials Research Center, Dental Faculty, Babol University of Medical Sciences, Babol, Iran.*; 2 *Cellular & Molecular Biology Research Center, Babol University of Medical Sciences, Babol, Iran.*; 3 *Non-communicable Pediatrics Diseases Research Center, Babol University of Medical Sciences, Babol, Iran.*; 4 *Students Research Committee, Babol University of Medical Sciences, Babol, Iran.*

**Keywords:** Oral lichen planus, angiogenesis, VCAM1, ICAM1

## Abstract

Oral lichen planus is a chronic inflammatory immune-mediated disease. ICAM-1 and VCAM-1 are vascular adhesion molecules that their receptors are located on endothelial cells and leukocytes. The aim of this study is the immunohistochemical evaluation of VCAM1 and ICAM1 in oral lichen planus and to compare these two markers with normal mucosa for evaluation of angiogenesis. This descriptive-analytical study was performed on 70 paraffined blocks of oral lichen planus and 30 normal mucosa samples taken from around the lesions. Samples were stained with H & E and then with Immunohistochemistry using monoclonal mouse anti human VCAM1 (CD106), & monoclonal mouse anti human ICAM1(CD54) for confirmation of diagnosis. Slides were evaluated under light microscope and VCAM1 and ICAM1 positive cells (endothelial cells and leukocytes) were counted. Data were analyzed with Mann-Whitney test, Wilcoxon and Chi-Square and p<0.001 was declared significant.

VCAM1 and ICAM1 expression significantly increased compared to normal mucosa in oral lichen planus according to the percentage of stained cells (p=0.000& p=0.000, Mann-Whitney test). Thirty cases of oral normal mucosa associated with lichen planus showed that the VCAM1 has increased significantly in comparison to normal mucosa (p<0.001). Also, ICAM1 expression between lichen planus and normal mucosa, showed a significantly difference (p<0.001). A significant difference between VCAM1 and ICAM1 expression and type of lichen planus was not observed (p>0.05). Regarding the results, it seems that high expression of VCAM1 and ICAM1 is related to oral lichen planus.

Lichen planus is a relatively common chronic inflammatory disease of the skin that mainly involves the oral mucosa. The disorder was named by the English physician Erasmus Wilson (1). With a prevalence of 1 to 2 percent, it has been indicated to be more frequent among females (2). Recent evidence suggests that the disease is an immune-mediated mucocutaneous disorder which is considered as a consequence of immune response to antigenic variations (3-6).

Lichen planus-triggering factor is unknown. Although it has been established that the presence of lymphocytes is necessary and increase in vascular adhesion molecules and cytokines are required for lymphocyte accumulation at a certain site, there is a supporting hypothesis indicating that the main mechanism for lichen planus is lymphocyte activation by increasing vascular adhesion factors, such as ELAM1, VCAM1, ICAM1, and lymphocytic infiltration through increase in L-selectin, LFA-1 and VLA4 receptors (7). Vascular adhesion factors are the proteins that provide interaction between leukocytes and endothelium. Studies have shown that the expression of adhesion molecules has been changed in oral lichen planus. VCAM1 and ICAM1 are vascular adhesion molecules that allow leukocytes to adhere to the vessel wall (8, 9).

Under normal conditions, small amount of ICAM1 is expressed by endothelial cells, monocytes and lymphocytes. Although ICAM1-induced cytokines are increased in the sites of inflammation, VCAM1 also mediates primary adhesion of leukocytes and their migration from blood vessels and this action is reinforced by ICAM1 (8, 9). VCAM1 (CD106) is one of the major vascular adhesion mediators directing the immune response (7, 10); it is composed of immunoglobulin chains that express in cytokine-stimulated large and small vessels (11). ICAM1 (CD54) is a single-chain glycoprotein on the surface of endothelial cells and immune system that stimulates immunological and inflammatory reactions (11, 12).

There is an increase in ICAM1 expression in inflammatory conditions with specific inflammatory mediators. This human antigen is observed in endothelial and other epithelial cells. ICAM1 binds to LFA1 and the latter, consisting of α and β subunits, is a member of the integrin family and a cell receptor; binding of ICAM1 and LFA1 can stimulate several reactions including T-cell specific responses to antigens as well as leukocytes binding to the endothelium and their migration (9, 13). It is believed that some of cytokines such as TNFα, IL-1 and IFN-α are responsible for increasing of vascular adhesion molecules. The source of these cytokines seems to be macrophages, factor Xllla-positive dendrocytes, Langerhans cells and lymphocytes in the inflammatory infiltrates of lichen planus (7).

Angiogenesis is a cycle of processes that eventuate in vascular anomalies in healthy vascular structures and is observed both physiologically and pathologically, occurring through endothelial cell activation and cytokine release (3); it plays a key role in chronic inflammatory diseases and leads to vessel sprouting, better oxygen delivery and turnover of the cells involved in inflammation (14). Angiogenesis includes protease release from endothelial cells and its migration into the interstitial space (15). Many studies have shown the role and the importance of angiogenesis in chronic inflammatory diseases. Blood vessel proliferation in oral lichen planus may be a response to the effect of hypoxia, meaning that lymphocyte proliferation is higher in places in which there is a response to the inflammation. VCAM1 and ICAM1 are considered the specific markers for the determination of endothelial alterations (3).

Regarding the fact that very limited studies have been conducted in the field of angiogenesis in lichen plaque, and only Scardina et al. investigation in 2007 and 2009 can be pointed out (3, 16), the necessity of further research on angiogenesis in lichen planus is stressed. Therefore, the present study was carried out to evaluate angiogenesis in lichen planus using immunohistochemical method.

## Materials and Methods

This descriptive-analytical study was implemented on paraffined blocks of 70 samples with lichen planus that were also clinically diagnosed according to WHO criteria in 2009, and 30 cases of normal mucosa surrounding the lesion obtained from the archives of oral and maxillofacial pathology department of Babol dental faculty during 2004-2012 (3). Five-micron sections were prepared from paraffined blocks and stained with H & E to confirm the diagnosis; proper cases with adequate length of epithelium were selected and 30 samples with normal mucosa at the periphery were chosen, regardless of patients' gender, for the study of normal mucosa. Immunohistochemical staining was performed with primary antibody, monoclonal mouse anti-human VCAM1 (CD106) (Abcam, UK) & monoclonal mouse anti-human ICAM1 (CD54) (Abcam, UK). Prepared microscopic slides were observed under light microscope (Olympus BX41, Tokyo, Japan) at 40× magnification, and positive cells in terms of VCAM1 and ICAM1 were randomly counted in five microscopic fields. The percentage of stained cells observed in the fields was scored as follows; score 0 for no staining, score 1 for stained cells less than 5% of cell population, score 2 for 5-25 %, and score 3 for stained cells more than 25% of cell population (17). Negative control was obtained with omission of primary antibody and positive control was placenta.

Mann-Whitney test was used to compare the markers expression between oral lichen planus and normal mucosa, and Wilcoxon test was applied for the difference between VCAM1 and ICAM1 expression in a lesion; Chi-Square test was also used to examine the relationship between the type of lichen planus (erosive and reticular) and VCAM1 and ICAM1 expression, and p<0.001 was considered significant.

## Results

**Fig 1:**
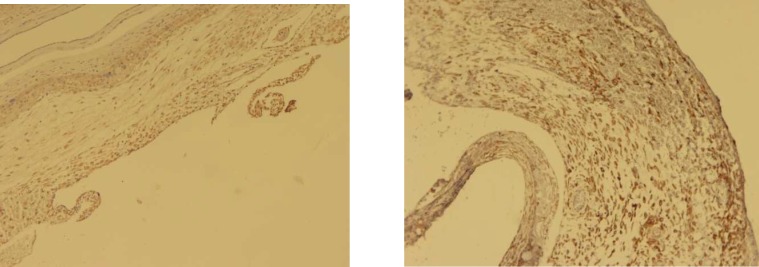
Immunohistochemical staining for VCAM1 and ICAM1 in positive control (placenta) 100x (right and left respectively

**Fig 2:**
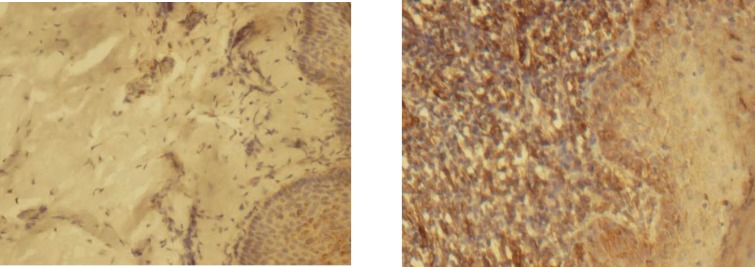
Immunohistochemical staining for VCAM1 in oral lichen planus and normal oral mucosa 400x (right and left respectively

**Fig 3:**
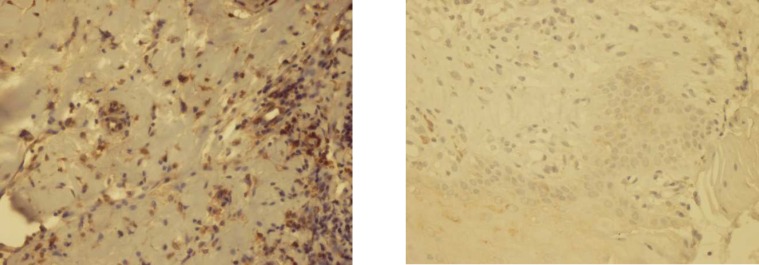
Immunohistochemical staining for ICAM1 in oral lichen planus and normal oral mucosa 400x (left and right respectively

In the present study, paraffined blocks of 70 patients with oral lichen planus (54 females and 16 males with the mean age of 44.91±1.488 years) and 30 samples of normal mucosa around the same lesions were used, and the results of immuno-histochemical staining for VCAM1 and ICAM1 expression in oral lichen planus and normal mucosa were examined and presented in Table 1 (Fig. 1, 2, 3). VCAM1 expression was evaluated by Wilcoxon test in 30 lesions and their surrounding normal mucosa. VCAM1 expression has been significantly more in lichen planus than normal mucosa (p<0.001). No case of higher or equal VCAM1 expression was observed in normal mucosa compared to lichen planus. Wilcoxon test also revealed higher ICAM1 expression in lichen planus compared to normal mucosa in 29 cases and in one case, ICAM1 was found to be equal in lichen planus and normal mucosa. In total, difference in ICAM1 expression was significant between lichen planus and normal mucosa (p<0.001).

**Table 1:** The results of immunohistochemical staining of oral lichen planus and normal mucosa for VCAM1 and ICAM1

Marker Score		Lichen planus	Normal mucosa	P value
VCAM1	Score 0 Score 1 Score 2 Score 3	0 (0%) 10 (14.3%) 28 (40%) 32 (45.7%)	30 (100%) 0 (0%) 0 (0%) 0 (0%)	0.000
ICAM1	Score 0 Score 1 Score 2 Score 3	0 (0%) 13 (18.5%) 15 (21.5%) 42 (60%)	28 (93.3%) 2 (6.7%) 0 (0%) (0%)	0.000

The highest VCAM1 and ICAM1 expression in lesions was observed for score 3; score 0 was found only in normal mucosa stained with VCAM1; however, for ICAM1, score 0 and 1 were observed. VCAM1 and ICAM1 expression was significantly more in lichen planus than normal mucosa. (p = 0.000, Mann-Whitney test).

Mann-Whitney test was used to compare the percentage of stained cells in oral lichen planus and normal mucosa, and the results showed significant increase in VCAM1 and ICAM1 expression in oral lichen planus compared to normal mucosa (p<0.001 and p<0.001). Mann-Whitney test was used to evaluate the association between the type of lichen planus (atrophic-erosive and reticular) and VCAM1 and ICAM1 expression; however, no significant difference has been found between the expression of these markers and the type of lichen planus (p=0.672 for VCAM1 and p=0.949 for ICAM1).

## Discussion

In the present study, VCAM1 and ICAM1 expression was significantly higher in lichen planus in comparison to normal mucosa. Regarding the fact that VCAM1 and ICAM1 are expressed by endothelial cells and leukocytes (8), the increase of their expression can be attributed to the increase in blood vessels and leukocytes in lichen planus. Therefore, it can be concluded that angiogenesis plays a major role in oral lichen planus, as an autoimmune disease with chronic inflammation basis, and the initiation of angiogenic response, either pathological or physiological, occurs through the activity of endothelial cells and cytokines release.

ICAM1 is a member of the immunoglobulin family that binds to integrin 2β, LFA1 and Mac1. LFA1 is expressed by almost all leukocytes in blood circulation and the reaction between ICAM1 and LFA1 has high importance in the adhesion and migration of leukocytes to the sites of inflammation. Stained blood vessels were observed in a normal mucosa exposed to ICAM1 in the study of Walton et al.; there was also an apparent increase in the number and intensity of vascular staining in oral lichen planus(4). Likewise, in the present study, a significant increase has been found in ICAM1 expression in lichen planus compared to normal mucosa, indicating the importance of ICAM1 in leukocyte migration from blood circulation to oral tissues.

VCAM1 is also a member of the immunoglobulin family that binds to integrin 1β and VLA4 and exists on the memory T-cells. In an immunohistochemical study, Walton et al. reported a slight expression of VCAM1 in endothelial cells of normal vessels in skin and a higher expression of which in inflammation of skin and dermatosis and observed a significant expression of VCAM1 in oral lichen planus. They also expressed that blood vessels stained with VCAM1 were higher in the vicinity of inflammatory infiltrate area than inside of the region (4). Scardina et al. also demonstrated the angiogenesis in oral lichen planus by immunohistochemical study using VCAM1, ICAM1, VEGF and CD34 (3). Similar to the present research, Dorrego et al. revealed a higher expression of VCAM1 and ICAM1 in oral lichen planus (8). According to Sangeetha et al., the expression of ELAM1, VCAM1, and ICAM1 is increased by endothelial cells of the subepithelial vascular network, and activated T-cells, mediated by VCAM1 and ICAM1, migrate to the oral epithelium (18).

However, Little et al. showed that in addition to the endothelial cells, ICAM1 is also expressed by oral mucosal keratinocytes in oral lichen planus, but not in normal oral mucosa (19).

Elevated VEGF has also been observed in the saliva of patients with oral lichen planus in Mittal et al.'s survey; VEGF secreted by macrophages and other cells of the immune system increases vascular proliferation and endothelial cell migration and is an important factor in the initiation and progression of angiogenesis which is severely raised in patients with oral lichen planus; they also stated that angiogenesis plays a significant role in the etio-pathogenesis and progression of lichen planus (20).

Lage et al. found an increase in ICAM1-expressing cells in lichen planus (21). In the current research, higher expression of ICAM1 has been detected in oral lichen planus, implicating its role in the inflammation and pathogenesis of the disease.

Oral lichen planus is an autoimmune disease caused by CD8+ T-cells that target the cells in basal layer. Accumulation of lymphocytes in the interface of the epithelium and the connective tissue occurs via an increase in cytokines and vascular adhesion molecules. There is an augmentation in the expre-ssion of vascular adhesion molecules including CD54 (ICAM1) and CD106 (VCAM1) in oral lichen planus in subepithelial vascular network which is induced by a number of cytokines such as TNFα, IL1 and IFN-γ produced by macrophages, Langerhans cells, lymphocytes and keratinocytes (22). On the other hand, Preissler et al. showed that ICAM1 inhibition can obviously lead to an increase in red blood cell flow and a decrease in adhesion and migration of leukocytes from the endothelium (23). The mentioned-above reasons can justify the increment in the number of leukocytes in superficial connective tissue in the form of band-like inflammatory infiltrates in lichen planus, so as increase in ICAM1 expression can be considered as a reason for an increased leukocytes number.

Evaluation of the expression of factors involved in angiogenesis may be an important step for the study of new therapies based on the use of anti-angiogenesis drugs which are currently admin-istered in other diseases with chronic inflammation pathogenesis and have been associated with good results. In addition, confirmation of the presence of angiogenesis in lichen planus can explain the cause of the disease which is still unknown. However, regarding the results of the present study and other similar investigations, it appears that the exclusive passage of lymphocytes from endothelial cells of the newly formed vessels and the presence of adhesion molecules in endothelial cells of these vessels can not only lead to oxygen supply to the tissues during the period of active disease but also to the renewal and sustainability of inflammatory components in the site of inflammation, thereby perpetuating the disease.

On the other hand, it can be stated that during the development of oral lichen planus, expression of vascular adhesion molecules such as CD54 (ICAM1) and CD106 (VCAM1) increases in the subepithelial vascular network, leading to lymphocyte accumulation in the interface of epithelium and connective tissue. Besides, cytokines produced by macrophages, Langerhans cells, lymphocytes and keratinocytes can increase the adhesion molecules. Vascular adhesion molecules allow leukocytes to adhere to the vessel wall. Although lichen planus-triggering factor is unknown, it has been established that the presence of lymphocytes is mandatory for the initiation of lichen planus. Therefore, the role of vascular adhesion molecules is suggested in the development of oral lichen planus.

In conclusion, higher expression of VCAM1 and ICAM1 were seen in oral lichen planus compared to normal oral mucosa which appears to be involved in pathogenesis of oral lichen planus. Moreover, evaluation of VCAM1 and ICAM1 in erosive and reticular lichen planus revealed no significant relationship between the type of lichen planus and the their expression.
